# A chiral three-dimensional network in poly[μ-4,4′-bipyridine-di-μ-formato-cadmium(II)]

**DOI:** 10.1107/S1600536809009969

**Published:** 2009-03-25

**Authors:** Li Zhao, Jian-Li Lin, Wei Xu, Hong-Zhen Xie

**Affiliations:** a State Key Laboratory Base of Novel Functional Materials and Preparation Science, Faculty of Materials Science and Chemical Engineering, Institute of Solid Materials Chemistry, Ningbo University, Zhejiang 315211, People’s Republic of China

## Abstract

In the title compound, [Cd(HCOO)_2_(C_10_H_8_N_2_)]_*n*_, the Cd^II^ ion, located on a position with 2.22 site symmetry, is surrounded by two 4,4′-bipyridine ligands and four formate ligands in a distorted octahedral CdN_2_O_4_ coordination. The 4,4′-bipyridine ligands bridge the metal ions, forming one-dimensional chains along different directions, which are further connected by formate ligands into a topologically (10^10^.12^4^.14)(10)_3_ three-dimensional network.

## Related literature

For the design and synthesis of coordination polymer complexes and their potential applications, see: Barbour (2006 ▶); Biradha (2003 ▶); Brammer (2004 ▶); Hosseini (2005 ▶); O’Keeffe & Yaghi (2001 ▶); Papaefstathiou & MacGillivray (2003 ▶); Venkataraman *et al.* (1995 ▶). For the 4,4′-bipyridine (4BPY) bridging ligand, see: Hagrman *et al.* (1999 ▶); Moulton & Zaworotko (2001 ▶); Sharma (2001 ▶); Zaworotko (2001 ▶). For one-dimensional zigzag networks using 2,2′-bpy as the ancillary ligand, see: Park *et al.* (2001 ▶). For the doubly inter­penetrated square grid network {[Zn(bipy)_2_(H_2_O)_2_][SiF_6_]}_*n*_, see: Subramanian & Zaworotko (1995 ▶). For a three-dimensional network with large channels constructed through square grid networks of 4BPY and Zn(II) linked by SiF_6_ anions, see: Gable *et al.* (1990 ▶).
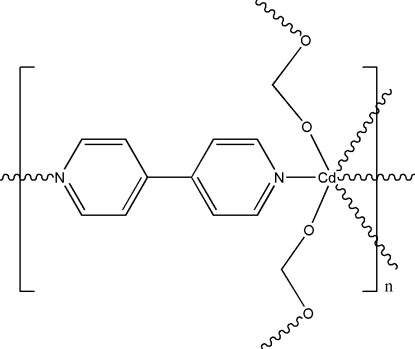


## Experimental

### 

#### Crystal data

[Cd(CHO_2_)_2_(C_10_H_8_N_2_)]
*M* *_r_* = 358.62
Tetragonal, 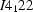

*a* = 8.2269 (12) Å
*c* = 18.103 (4) Å
*V* = 1225.2 (4) Å^3^
*Z* = 4
Mo *K*α radiation
μ = 1.79 mm^−1^
*T* = 293 K
0.33 × 0.33 × 0.20 mm

#### Data collection

Rigaku R-AXIS RAPID diffractometer
Absorption correction: multi-scan (*ABSCOR*; Higashi, 1995 ▶) *T*_min_ = 0.554, *T*_max_ = 0.698
1106 measured reflections
711 independent reflections
681 reflections with *I* > 2σ(*I*)
*R*_int_ = 0.025

#### Refinement

*R*[*F* ^2^ > 2σ(*F* ^2^)] = 0.018
*wR*(*F* ^2^) = 0.046
*S* = 1.13
711 reflections
46 parameters
H-atom parameters constrained
Δρ_max_ = 0.21 e Å^−3^
Δρ_min_ = −0.43 e Å^−3^
Absolute structure: Flack (1983 ▶), 263 Friedel pairs
Flack parameter: 0.02 (7)

### 

Data collection: *RAPID-AUTO* (Rigaku, 1998 ▶); cell refinement: *RAPID-AUTO*; data reduction: *CrystalStructure* (Rigaku/MSC, 2002 ▶); program(s) used to solve structure: *SHELXS97* (Sheldrick, 2008 ▶); program(s) used to refine structure: *SHELXL97* (Sheldrick, 2008 ▶); molecular graphics: *SHELXL97*; software used to prepare material for publication: *SHELXL97*.

## Supplementary Material

Crystal structure: contains datablocks global, I. DOI: 10.1107/S1600536809009969/jh2074sup1.cif

Structure factors: contains datablocks I. DOI: 10.1107/S1600536809009969/jh2074Isup2.hkl

Additional supplementary materials:  crystallographic information; 3D view; checkCIF report

## Figures and Tables

**Table 1:** Selected geometric parameters (Å, °)

Cd1—N1	2.306 (3)
Cd1—N1^i^	2.306 (3)
Cd1—O1^ii^	2.3264 (18)
Cd1—O1^i^	2.3264 (18)
Cd1—O1^iii^	2.3264 (18)
Cd1—O1	2.3264 (18)

Symmetry codes: (i) 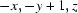; (ii) 
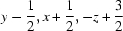
; (iii) 
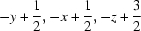
.

**Fig. 1.**
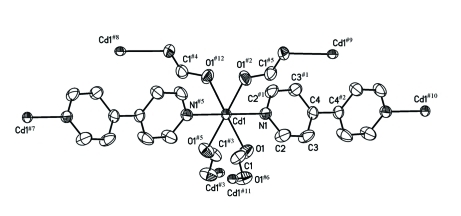
A view of the complex molecule of the title compound, showing the atom-numbering scheme. Displacement ellipsoids are drawn at the 60% probability level [Symmetry codes: #1 = -x + 1/2, -y + 1/2, -z + 3/2; #2 = -x + 1, -y, z; #3 = x + 1/2, -y + 1, z + 1/4; #4 = -x + 1/2, y - 1, z + 1/4; #5 = -x, -y + 1, z; #6 = x, -y + 2/3, -z + 5/4; #7 = x - 1, y + 1, z; #8 = x + 1/2, -y, z + 1/4; #9 = x, -y + 1/2, z - 1/4; #10 = x + 1, y - 1, z; #11 = x + 1, -y + 1/2, z - 1/4; #12 = 1 - x, -y, z].

**Fig. 2.**
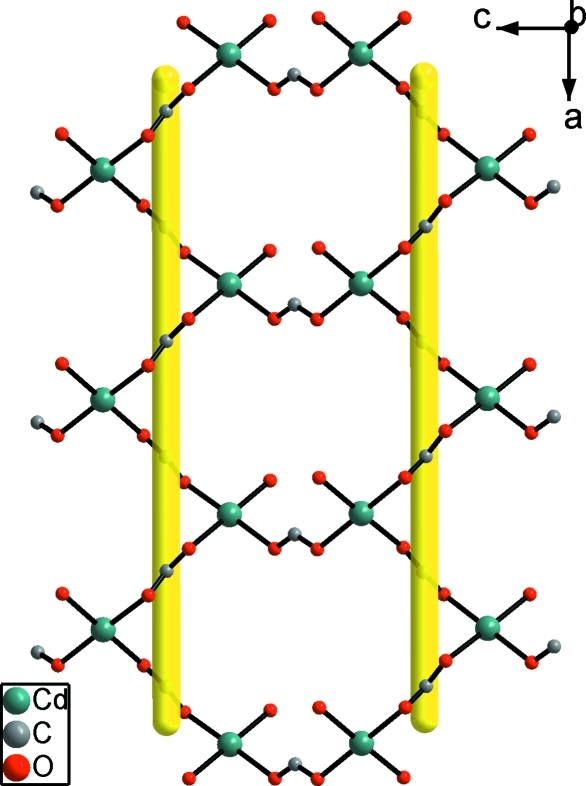
Right-handed one-dimensional helical chains along a axis.

**Fig. 3.**
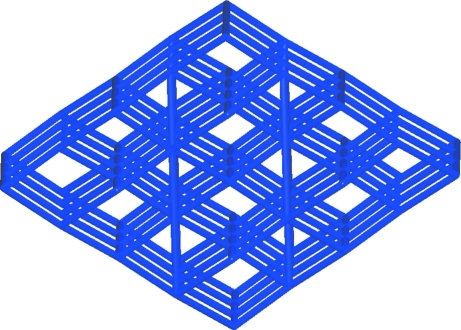
Topological representation of the three-dimensional structure.

## supplementary crystallographic information

### Comment

The design and synthesis of coordination polymer complexes, which is an emerging area of research with several potential applications in areas such as catalysis, conductivity, porosity, chirality, luminescence, magnetism, spin-transition and non-linear optics, are of considerable interest from the viewpoint of crystal engineering (Barbour, 2006; Biradha, 2003; Brammer, 2004; Hosseini, 2005; O'Keeffe, 2001; Papaefstathiou, 2003; Venkataraman, 1995). The structures and properties of coordination polymers can be controlled by choosing appropriate bridging ligands and metal ions. Many types of bridging ligand have been reported, of which the most extensively studied bidentate ligands are probably 4,4'-bipyridine (4BPY) (Hagrman, 1999; Moulton, 2001; Sharma, 2001; Zaworotko, 2001). The ligand 4,4'-bipyridine is an ideal connector between the transition metal atoms for the propagation of coordination networks and shown to form a variety of networks ranging from one-dimensional to three-dimensional with several transition metal salts, such as the one-dimensional zigzag networks by using of 2,2'-bpy as the ancillary ligand (Park, 2001), the doubly interpenetrated square grid networks {[Zn(bipy)_2_(H_2_O)_2_][SiF_6_]}_n_ (Gable, 1990) and a three-dimensional network with large channels which is constructed through square grid networks of 4BPY and Zn(II) linked by SiF_6_ anions (Subramanian, 1995). In this contribution, we here report a novel chiral three-dimensional crystal structure built from Cd^II^ ions and mixed-ligand including 4BPY ligands and formic acid ligands.

Compound 1 consists of Cd^II^ ions, 4,4'-bipyridine (4BPY) ligands and formato anions. As illustrated in Figure 1, the Cd^II^ ions are all disposed in a N_2_O_4_ octahedron coordination environment with the equatorial coordination from four formate ligands (O1, O1^#2^, O1^#5^, O1^#12^) [symmetry codes: #2 = 1 - *x*, -*y*, *z*; #5 = -*x*, 1 - *y*, *z*; #12 = 1 - *x*, -*y*, *z*] and the apical sites occupied by two N atoms from two 4BPY ligands (N1, N1^#5^) [symmetry code: #5 = -*x*, 1 - *y*, *z*]. The average bond lengths of Cd—O and Cd—N are 2.326 (3) Å and 2.306 (4) Å, respectively. There are two kinds of formate ligands in this sturcture, one kind of them connect metal ions into right-handed helical chains along *a* axis (Figure 2) and the others connecte the helical chains along two different orientations [011] and [011], which generate a three-dimensional network. In the resulting structure, 4BPY ligands further link the Cd^II^ ions along [110] and [110] directions,respectively, to generate a (10^10^.12^4^.14)(10)_3_ topological three-dimensional network (Figure 3). Moreover, the 4BPY ligand displays obvious distortion and the dihedral angel between the two pyridine rings is approximately 46.2°.

### Experimental

4,4'-bpy (0.153 g, 1.0 mmol) and CdCO_3_ (0.177 g, 1.0 mmol) were orderly added in 10 ml H_2_O. Under continous stirring, 5.5 ml HCOOH (2 *M*) solution was subsequently added in the resulting mixture yielding clear solution. After filtration, the filtrate was maintained for slow evaporation at 40°C constant temperature for 3 days. Light yellow granule-liked crystals were obtained in a yield of *ca* 23.7% based on CdCO_3_.

### Refinement

H atoms bonded to C atoms were palced in geometrically calculated position and were refined using a riding model, with *U*_iso_(H) = 1.2 *U*_eq_(C). H atoms attached to O atoms were found in a difference Fourier synthesis and were refined using a riding model, with the O—H distances fixed as initially found and with *U*_iso_(H) values set at 1.2 *U*eq(O).

### Crystal data

**Table:**

[Cd(CHO_2_)_2_(C_10_H_8_N_2_)]	*D*_x_ = 1.944 Mg m^−^^3^
*M**_r_* = 358.62	Mo *K*α radiation, λ = 0.71073 Å
Tetragonal, *I*4_1_22	Cell parameters from 1106 reflections
Hall symbol: I 4bw 2bw	θ = 3.2–27.5°
*a* = 8.2269 (12) Å	µ = 1.79 mm^−^^1^
*c* = 18.103 (4) Å	*T* = 293 K
*V* = 1225.2 (4) Å^3^	Granule, yellow
*Z* = 4	0.33 × 0.33 × 0.20 mm
*F*(000) = 704	

### Data collection

**Table:**

Rigaku R-AXIS RAPID diffractometer	711 independent reflections
Radiation source: fine-focus sealed tube	681 reflections with *I* > 2σ(*I*)
graphite	*R*_int_ = 0.025
Detector resolution: 0 pixels mm^-1^	θ_max_ = 27.5°, θ_min_ = 2.7°
ω scans	*h* = −10→1
Absorption correction: multi-scan (*ABSCOR*; Higashi, 1995)	*k* = −10→1
*T*_min_ = 0.554, *T*_max_ = 0.698	*l* = −23→1
1106 measured reflections	

### Refinement

**Table:**

Refinement on *F*^2^	Secondary atom site location: difference Fourier map
Least-squares matrix: full	Hydrogen site location: inferred from neighbouring sites
*R*[*F*^2^ > 2σ(*F*^2^)] = 0.018	H-atom parameters constrained
*wR*(*F*^2^) = 0.046	*w* = 1/[σ^2^(*F*_o_^2^) + (0.018*P*)^2^ + 0.9631*P*] where *P* = (*F*_o_^2^ + 2*F*_c_^2^)/3
*S* = 1.13	(Δ/σ)_max_ < 0.001
711 reflections	Δρ_max_ = 0.21 e Å^−^^3^
46 parameters	Δρ_min_ = −0.43 e Å^−^^3^
0 restraints	Absolute structure: Flack (1983), 263 Friedel pairs
Primary atom site location: structure-invariant direct methods	Flack parameter: 0.02 (7)

### Special details

**Table:**

Geometry. All e.s.d.'s (except the e.s.d. in the dihedral angle between two l.s. planes) are estimated using the full covariance matrix. The cell e.s.d.'s are taken into account individually in the estimation of e.s.d.'s in distances, angles and torsion angles; correlations between e.s.d.'s in cell parameters are only used when they are defined by crystal symmetry. An approximate (isotropic) treatment of cell e.s.d.'s is used for estimating e.s.d.'s involving l.s. planes.
Refinement. Refinement of *F*^2^ against ALL reflections. The weighted *R*-factor *wR* and goodness of fit *S* are based on *F*^2^, conventional *R*-factors *R* are based on *F*, with *F* set to zero for negative *F*^2^. The threshold expression of *F*^2^ > σ(*F*^2^) is used only for calculating *R*-factors(gt) *etc*. and is not relevant to the choice of reflections for refinement. *R*-factors based on *F*^2^ are statistically about twice as large as those based on *F*, and *R*- factors based on ALL data will be even larger.

### Fractional atomic coordinates and isotropic or equivalent isotropic displacement parameters (Å^2^)

**Table:**

	*x*	*y*	*z*	*U*_iso_*/*U*_eq_	
Cd1	0.0000	0.5000	0.7500	0.02047 (10)	
O1	0.1521 (3)	0.6479 (3)	0.66505 (10)	0.0380 (4)	
N1	0.1982 (2)	0.3018 (2)	0.7500	0.0284 (6)	
C1	0.0885 (5)	0.7500	0.6250	0.0354 (9)	
H1A	−0.0282	0.7500	0.6250	0.042*	
C2	0.3461 (3)	0.3320 (3)	0.72483 (18)	0.0431 (7)	
H2	0.3680	0.4350	0.7062	0.052*	
C3	0.4696 (3)	0.2183 (3)	0.7249 (2)	0.0432 (8)	
H3	0.5731	0.2454	0.7083	0.052*	
C4	0.4359 (3)	0.0641 (3)	0.7500	0.0269 (7)	

### Atomic displacement parameters (Å^2^)

**Table:**

	*U*^11^	*U*^22^	*U*^33^	*U*^12^	*U*^13^	*U*^23^
Cd1	0.01803 (12)	0.01803 (12)	0.02535 (16)	0.00289 (14)	0.000	0.000
O1	0.0320 (11)	0.0393 (12)	0.0428 (10)	0.0038 (8)	0.0064 (10)	0.0191 (10)
N1	0.0214 (8)	0.0214 (8)	0.0425 (15)	0.0042 (10)	0.0017 (10)	0.0017 (10)
C1	0.0238 (19)	0.047 (2)	0.0354 (19)	0.000	0.000	0.0116 (18)
C2	0.0256 (13)	0.0257 (13)	0.078 (2)	0.0063 (9)	0.0064 (14)	0.0173 (14)
C3	0.0197 (14)	0.0333 (14)	0.077 (2)	0.0050 (11)	0.0072 (12)	0.0180 (14)
C4	0.0228 (9)	0.0228 (9)	0.0352 (16)	0.0083 (13)	−0.0012 (12)	−0.0012 (12)

### Geometric parameters (Å, °)

**Table:**

Cd1—N1	2.306 (3)	C1—O1^iv^	1.227 (3)
Cd1—N1^i^	2.306 (3)	C1—H1A	0.9600
Cd1—O1^ii^	2.3264 (18)	C2—C3	1.381 (4)
Cd1—O1^i^	2.3264 (18)	C2—H2	0.9300
Cd1—O1^iii^	2.3264 (18)	C3—C4	1.376 (3)
Cd1—O1	2.3264 (18)	C3—H3	0.9300
O1—C1	1.227 (3)	C4—C3^iii^	1.376 (3)
N1—C2	1.323 (3)	C4—C4^v^	1.492 (7)
N1—C2^iii^	1.323 (3)		
			
N1—Cd1—N1^i^	180.0	C2—N1—C2^iii^	117.6 (3)
N1—Cd1—O1^ii^	90.61 (6)	C2—N1—Cd1	121.20 (16)
N1^i^—Cd1—O1^ii^	89.39 (6)	C2^iii^—N1—Cd1	121.20 (16)
N1—Cd1—O1^i^	90.61 (6)	O1—C1—O1^iv^	129.5 (4)
N1^i^—Cd1—O1^i^	89.39 (6)	O1—C1—H1A	115.3
O1^ii^—Cd1—O1^i^	178.78 (12)	O1^iv^—C1—H1A	115.3
N1—Cd1—O1^iii^	89.39 (6)	N1—C2—C3	123.4 (3)
N1^i^—Cd1—O1^iii^	90.61 (6)	N1—C2—H2	118.3
O1^ii^—Cd1—O1^iii^	97.24 (10)	C3—C2—H2	118.3
O1^i^—Cd1—O1^iii^	82.77 (10)	C4—C3—C2	118.4 (3)
N1—Cd1—O1	89.39 (6)	C4—C3—H3	120.8
N1^i^—Cd1—O1	90.61 (6)	C2—C3—H3	120.8
O1^ii^—Cd1—O1	82.77 (10)	C3^iii^—C4—C3	118.7 (3)
O1^i^—Cd1—O1	97.24 (10)	C3^iii^—C4—C4^v^	120.63 (16)
O1^iii^—Cd1—O1	178.78 (12)	C3—C4—C4^v^	120.63 (16)
C1—O1—Cd1	121.3 (2)		

Symmetry codes: (i) −*x*, −*y*+1, *z*; (ii) *y*−1/2, *x*+1/2, −*z*+3/2; (iii) −*y*+1/2, −*x*+1/2, −*z*+3/2; (iv) *x*, −*y*+3/2, −*z*+5/4; (v) −*x*+1, −*y*, *z*.
